# Supplementary figures and images for: A New Sensory Approach Combined with a Text-Mining Tool to Create a Sensory Lexicon and Profile of Monovarietal Apple Juices
Source: Foods. 2019 Nov 22;8(12):608. doi: 10.3390/foods8120608 (PMC6963934; doi:10.3390/foods8120608)

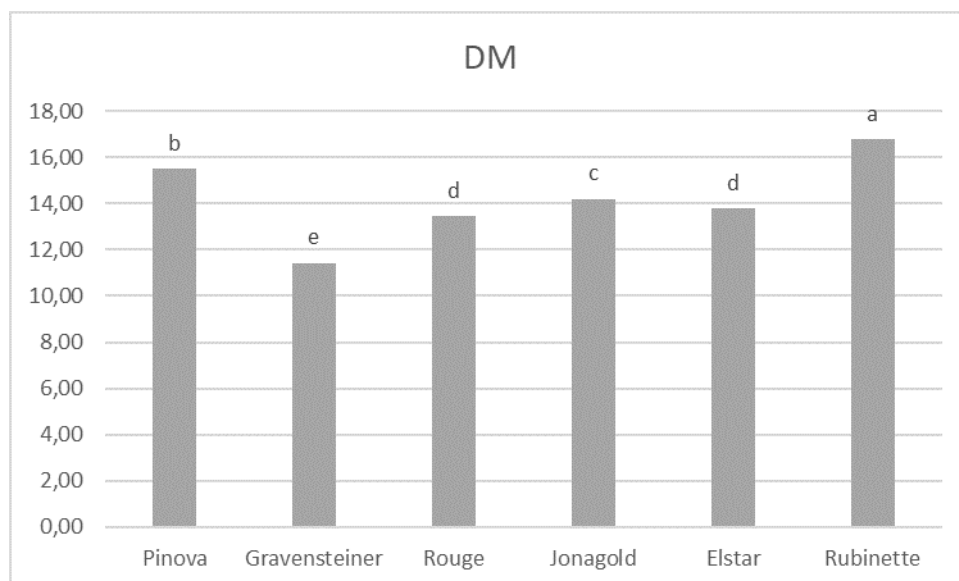

**Figure S1.** Results of dry matter were consistent with TSS values for all samples.

Supplement: Supplementary file 1 [file foods-08-00608-s001.pdf]
